# The Result of Twenty-two Doses of Chloral Hydrate; with Cases, Observations and Gleanings, at St. Luke’s Hospital

**Published:** 1870-11

**Authors:** John E. Owens

**Affiliations:** Surgeon to the Hospital


ARTICLE II.—*The Result of Twenty-two Doses of Chloral Hydrate; with Cases, Observations and Gleanings, at St. Luke's Hospital.* By JNO. E. OWENS, M.D., Surgeon to the Hospital.

Dose 1, Case A. W. R., aged 47, in the last stage of phthisis, has been taking Morph. Sulph. gr. i; *pro re nata.* Last evening, (May 11th), being very restless, and in much pain and general distress, at 6 o'clock, Chloral Hydrate, grs. xx, were given. Within about fifteen or twenty minutes he began to doze, and at a quarter to 7 o'clock he was in a profound sleep. The respiration was not so frequent as when he was awake, the chin dropped, the eye-balls were only half covered, pinching occasioned no response, the arms became cold to the elbows; he could not be aroused, and remained in this condition about two hours, after which he awoke, sucked a piece of orange, fell asleep again, and remained so, awaking for a moment once or twice, till 4 o'clock in the morning.

Dose 2, Case A. The resident, being somewhat timid, in consequence of the effect of the first dose, gave at 10 A. M., (May 12th), only 15 grs., which produced a calmness.

Dose 3, Case A. At 7 o'clock, P. M., 20 grs. produced sound sleep, but the patient was able to be aroused.

Dose 4, Case A. Chloral Hydrate, 20 grs., at 10 o'clock, A. M., (May 14th), caused drowsiness in 15 minutes. Patient died, May 17th.

Dose 5, Case B. May 18th, a young, delicate woman was admitted with typhoid fever, complicated with unusual cerebral excitement. She talked more or less incoherently, jumped from bed five or six times during the night, and as frequently through the day. At half past 7 P. M., the patient being wild and excitable, but easily managed, took Chloral Hydrate, 20 grs. In 15 minutes she became calm, in a half hour slept soundly, and passed a very quiet night.

Dose 6, Case B. May 19th, during the morning she was pretty quiet, and more or less drowsy, no inclination to get up. About 12 M., the patient becoming again restless, we gave Chloral Hydrate, 20 grs. She was asleep at 12.15, and did not get up during the night. Twice, however, she asked for water, and after drinking dropped off to sleep again.

Dose 7, Case B. May 21st, last evening 20 grs. caused sleep in five minutes by the watch. In ten minutes, so soundly asleep was she that the resident lifted her hand from the bed, felt the pulse and dropped the hand upon the patient's body without awaking her. Previous to the administration of the medicine she was wakeful, and staring wildly around the room.

Dose 8, Case B. In fifteen minutes after another dose (20 grs.) she became quiet, and remained so for two or three hours, after which the effect of the medicine had entirely passed away.

Dose 9, Case B. At 3 o'clock, P. M., the dose was repeated. It took effect in 15 minutes, but its influence continued only 45 minutes.

Dose 10, Case B. May 31st, patient being wakeful and nervous, we gave 20 grs. at 8 P. M. She was asleep in 25 minutes, and slept nearly all night.

Dose 11, Case C. A large muscular man was admitted with stricture of the urethra. A day or two after admission, Chloral Hydrate, 20 grs., were given. The patient was not suffering any at the time. He was put to bed without supper, in a quiet ward, but did not sleep, and said that he did not feel drowsy. Dose was insufficient.

Dose 12, Case D. J. Q., aged 23, was admitted for partial anchylosis of the knee, the result of synovitis. There was slight dullness at the apex of the right lung, laryngitis supervened, the patient was feverish, and could not speak above a whisper. At half past 2 P. M., Chloral Hydrate, 20 grs., were taken, the patient being in bed. In 20 minutes he became excited. The excitement, which continued about 10 minutes, was similar to that produced by chloroform. This excitement was manifested by the patient's throwing his arms about, and by his expressing a desire to fight. Falling asleep in 35 minutes, he awoke in an hour, but in a few moments he went to sleep again and remained so for two hours.

Dose 13, Case E. A tall man with large frame and large joints, having been admitted, several days ago, with acute rheumatism, took Chloral Hydrate, 20 grains; negative result; dose insufficient.

Dose 14, Case E. Chloral Hydrate, 25 grains, were administered; negative result; dose insufficient.

Doses 15 and 16, Case F. A tall, stout young man was admitted on account of persistent hæmorrhage from the arm—the consequence of a stab received, in New Orleans, two months before admission. Upon cutting down close to the bleeding vessel, and applying the actual cautery, the bleeding ceased. The night of the operation he suffered much pain, for which he took Morph. Sulph. grs. ss., with negative result, although the pain was somewhat lessened. The second night, though the pain was not so great as that of the preceding one, Chloral Hydrate, 20 grs., were given, at 8 o'clock P. M. We waited an hour and a half, and seeing no effect from the dose, 40 grs. were administered. In thirty minutes he was asleep, and passed a very comfortable night, having been disturbed only to go to stool. He felt well in the morning, there being no headache, or nausea, and he ate a hearty breakfast.

Dose 17, Case F. Hand considerably swollen and very painful. At 11 o'clock last night, gave Chloral Hydrate, 40 grs. The patient was watched an hour. In 15 or 20 minutes he ceased to groan, and was sleeping soundly at the expiration of the hour. At the end of two hours he called the resident, telling him that the hand and arm were again painful. He very soon went to sleep, however, and passed the balance of the night in comfort.

Dose 18, Case G. A well developed and stout male patient, aged 23, above the medium height, was operated upon to-day (May 6th) for the relief of bony anchylosis of the elbow. For pain after the operation, the patient took Morph. Sulph. 1-4 gr., at 6 P. M.; negative result. At 7 P. M., the resident gave Morph. Sulph. gr. ss., a half hour after which he went to sleep and slept an hour, and then awoke with pain. At 9 P. M., we gave Chloral Hydrate, 30 grs. Ten minutes after taking this dose, he went to sleep, and continued to sleep well during the night. This morning, (Aug. 7th), upon awakening he felt well, had some appetite for breakfast, after which he occupied himself in reading.

Dose 19, Case G. Aug. 8th, at 9 P. M., the patient suffering from headache and pain in the arm, took Chloral Hydrate, grs. xxv, with negative result, both as to decrease of pain and the production of sleep.

Doses 20, 21 and 22, Case H. Dr. Hudson (Aug. 8th), the resident, aged 24, well developed and healthy, took Chloral Hydrate, grs. xx, at half past 10 o'clock A. M. At 11 A. M., he had experienced no change of feeling. At this time (11 A. M.) he repeated the dose, (grs. xx), ten minutes after which, he was somewhat excited, and experienced a warm, glowing sensation over the whole body, but more especially about the head and chest. The sensation was agreeable. In 20 minutes, the excitement was at its acme. He felt like one slightly intoxicated. At 11.30 A. M., being extremely happy, and concluding "to make a little heaven here on earth," he took a third dose, (grs. xxx). Five minutes after taking the last dose, the inclination to lie down was irresistible. Acting accordingly, he did not remember anything after touching the sofa, till 1.30 o'clock P. M., when some one aroused him for dinner. Both bells had rung, the patients had passed and repassed the office door, on their way to and from dinner, without awaking him. He went to dinner, feeling as well as usual, and ate as heartily as he had done for a month. There were no after effects whatever. (The notes of Case F are almost entirely in Dr. Hudson's own words, taken from a letter written after he left the hospital.)

Ever on the watch for remedial agents to mitigate human suffering, both the surgeon and physician must hail with delight this new and fascinating hypnotic anodyne. Surer than opium and its salts, and unaccompanied by their unpleasant consequences—constipation, loss of appetite, profuse perspiration, mental hebetude, nausea and vomiting—Chloral Hydrate seems to be rapidly approaching a position unattained by any other drug of its class. Opium, for centuries the idol of the physician, the sheet anchor of the surgeon, and a blessing to mankind, has now a powerful rival. Discovered (Chloral) by Liebig, probably more than twenty years ago, it was first employed as a hypnotic and anæsthetic by Liebreich, of Berlin. Chloral is a colorless fluid, which should never be used in practice. Upon being mixed with water, however, it becomes the Hydrate—a white, solid substance, having an odor resembling that of ripe melons, and a somewhat caustic taste. It is the latter, in solution, that is used in practice. M. Personne's communications on Chloral, read before the Academy of Sciences, of Paris, point to its alternate transformation in the organism into formed acid and Chloroform, the latter being subsequently converted into Chloride of Sodium and Formiate of Soda—the products of its elimination. It is the alkali, alone, used in these experiments, which transforms Chloral into Chloroform. The theory of action, then, is that by the influence of the alkaline reaction of the blood, Chloral is changed into Chloroform, which produces sleep and sometimes anæsthesia. The sleep is more slowly produced than by Chloroform, lasts longer, and is not so frequently preceded by excitement. Chloral is most conveniently administered by the mouth. A much smaller dose is required by the hypodermic method, but as the dose that will kill by this method, has not, as yet, been determined, its administration in this manner is considered unsafe. M. Wamias, however, a surgeon of Venice, has not experienced any inconvenience after hypodermic injections of Chloral, and says that 15 grs., in double the quantity of distilled water, have acted speedily and excellently. This remedy seems to be contraindicated in the same cases in which Chloroform is contraindicated, namely, in organic disease of the heart and brain. Dr. Richardson, I believe, recognizes only one form of organic heart disease, in which Chloroform is positively contraindicated, namely, a heart with its right ventricle dilated, and attended with bronchial cough and dilated veins. It is impossible to fix a uniform dose of Chloral. It ranges from ten to eighty grains. It is safe, in the adult, to begin with 20 grs., and increase five or ten grains *pro re nata.* I have seen very good results, in cases of pulmonary irritation, from ten grains three times daily. The dose is, in a degree, proportionate to the size and strength of the patient; a strong, vigorous man, above the medium height, requiring from ten to twenty grains more than one of ordinary strength and medium size. Liebreich may have entertained a hope of its rivaling Chloroform, Ether, and other anæsthetics, as used in surgery, but for this purpose Chloral is totally uncertain and dangerous. Any attempt to induce anæsthesia by the latter drug is wholly unjustifiable. It, however, promptly produces a stupor and sleep, which extends over considerable time with safety. It is used for sustaining sleep in the same class of cases for which Opium, Hemlock, Hyoscyamus, and Belladonna are commonly used, and we need only glance at the above cases in order to see how promptly and reliably it acts, and how free it is from uncomfortable after effects.

Doses 1, 2, 3 and 4, acted in fifteen to twenty minutes; doses 5 and 6, acted in fifteen minutes; dose 7, in five minutes; doses 8 and 9, in from fifteen to twenty-five minutes.

Dose 11, Case C. Nil. The patient was a large and strong man, requiring more than 20 grs.

Dose 12, caused excitement in twenty minutes, and sleep in thirty-five minutes.

Doses 13 and 14. Nil. A large man, requiring more than 25 grs.

Dose 15, Case F. Nil. A tall, stout man, requiring more than 20 grs.; but dose 16 (40 grs.) in case F, was followed by sleep in thirty minutes.

Dose 18, Case G. (30 grs), acted in ten minutes, after Morphia had, to a great extent, failed.

The most interesting case, that of Dr. Hudson, (doses 20, 21 and 22,) will speak for itself. None of these doses were followed by constipation, loss of appetite, profuse perspiration, mental hebetude, nausea or vomiting. After several doses there was moderate gastric burning; after two doses, anæsthesia. The same care must be exercised in the administration of this remedy, as in other medicines of its class. Highly dangerous symptoms of depression have been produced by a dose of 50 grs. Dr. Post has told me of similar cases happening in New York. Where Chloral is impure it is without action, and is considered dangerous. Very recently we had a bottle of Chloral Hydrate that was moist and sticky. It was here and there of a sulphur yellow, especially the thinner masses that adhered to the inside of the bottle. With this specimen we never had other than a negative result, although it was used in doses as high as eighty grains. After examining the specimen, we of course discontinued its use. Again, the preparations of Chloral Hydrate should not be kept on hand too long previous to use, as they may, in this way, lose their efficacy. Liebreich, after some experiments, concludes that Strychnine, after a too heavy dose of Chloral, shortens and destroys the effect of the remedy, and without producing the injurious action which it exhibits in ordinary cases. He, therefore, proposes to employ hypodermic injections of Nitrate of Strychnine, as an antidote, in cases of the occurrence of accidents after an overdose of Chloral or Chloroform. M. Verneuil has presented a note to the French Academy of Sciences, in which he says that experiments having established the fact that Chloral is antagonistic of Strychnine, it might prove useful in tetanus. Subsequently, Liebreich reported a case of rapid recovery of this affection by the agency of Chloral Hydrate. Another case has occurred in Lariboisière Hospital, of complete recovery. The daily dose ranged from ℨ iss. to ℨ ij. There is also a third cure reported.

In the management of the nervous, Chloral Hydrate is an agent of great value. In a case of puerperal mania* in which Bailley's solution of Opium and Potass. Bromid. had failed, Chloral, (30 to 60 grs.) produced, in a few minutes, a complete cessation of all cries and struggles, and a return of calm consciousness, followed by a natural sleep of six or eight hours, and this probably saved the life of the patient. In the agitation which sometimes follows anæsthesia from Chloroform, Chloral

* Lancet.

Hydrate produces very happy effects. It is a calmer of general irritability, and an invaluable remedy in the treatment of neuralgia and delirium tremens. For neuralgia of some of the ramifications of the fifth nerve, we have found 20 grs., in the case of rather delicate females, act very promptly. In delirium tremens 40 grs. is a fair dose. It is said that, in children, about six years old, suffering from pertussis, 5 grs., two or three times daily, will mitigate the paroxysms; or, if the paroxysms are more severe at night, that six grains, at bed-time, will ensure a quiet night. Prof. Gross recommends it, in the treatment of strangulated hernia. Indeed, were we to enumerate all the affections or conditions for which Chloral Hydrate may be used with great advantage, we would encroach too much, perhaps, upon the pages of the JOURNAL. We hope, however, that a medicine so valuable will very soon be offered to the profession at cheaper rates, provided this can be done without any deterioration of quality, for Chloral that is not perfectly good and pure, is simply worth nothing.

